# Elucidate senescence-related gene signature and immune infiltration landscape in abdominal aortic aneurysm

**DOI:** 10.1371/journal.pone.0340976

**Published:** 2026-01-20

**Authors:** Jingde Li, Ru Ying, Jing Luo, Xin Guo, Min Zhang

**Affiliations:** 1 Department of Cardiology, The Central Hospital of Wuhan, Tongji Medical College, Huazhong University of Science and Technology, Wuhan, China; 2 Key Laboratory for Molecular Diagnosis of Hubei Province, The Central Hospital of Wuhan, Tongji Medical College, Huazhong University of Science and Technology, Wuhan, China; 3 Department of Cardiology, The First Affiliated Hospital of Nanchang University, Nanchang, China; 4 Jiangxi Hypertension Research Institute, Nanchang, China; Army Medical University, CHINA

## Abstract

**Background:**

Abdominal aortic aneurysm (AAA) refers to a lasting enlargement of the abdominal aorta. Senescence, a major risk factor of AAA, demonstrate positive connection with both the formation and rupture of aneurysms. Therefore, investigating the underlying pathogenic mechanisms of senescence in AAA and exploring relevant diagnostic and therapeutic targets is crucial.

**Methods:**

Three transcriptomic datasets related to AAA were obtained from the GEO database, and collection of genes associated with cellular senescence was obtained from MSigDB. Overlapping genes of differentially expressed genes (DEGs), module genes associated with AAA, and senescence-related gene sets were identified as senescence-related DEGs of AAA and subjected to further functional enrichment analysis. Distinct machine learning algorithms were subsequently utilized to screen for senescence-associated biomarkers and develop a diagnostic nomogram. In addition, the interaction between these biomarkers and immune components in the aneurysmal environment were revealed. Consensus clustering was subsequently applied to classify AAA into distinct subtypes. Finally, validation was performed using an AAA murine model.

**Results:**

A total of 11 senescence-related DEGs in AAA were identified, which mainly involved with oxidative stress, inflammatory responses, and vascular smooth muscle cell activity. Following rigorous screening, IL6, ETS1, TDO2, and TBX2 were identified as diagnostic biomarkers for senescence-related DEGs of AAA. The nomogram constructed from these biomarkers demonstrated high discriminatory ability in the training cohort (AUC = 1), though this requires further validation in larger cohorts due to potential overfitting. Immune cell infiltration and single-cell analyses indicated that the expression of the diagnostic biomarkers is linked to various immune cell types. Consensus clustering identified two AAA subtypes, which exhibiting distinct expression patterns of senescence-related biomarkers. Finally, validation in an AAA murine model confirmed the expression changes of these senescence-related biomarkers in AAA.

**Conclusion:**

This study identified senescence-related biomarkers associated with AAA through transcriptomic public databases, revealing their potential functional mechanisms, relationships with immune cells, and associations with AAA subtypes. These results could offer novel candidate targets for both diagnostic and therapeutic strategies in AAA.

## 1. Introduction

Abdominal aortic aneurysm (AAA) is clinically referred as a focal enlargement of the abdominal aorta measuring more than 3 cm in diameter or surpassing 50% of its typical width [1]. This condition is typically asymptomatic; however, once an AAA ruptures, it leads to severe hemorrhage and carries an exceedingly high mortality rate [2]. Although aging, male gender, abnormal lipid profiles, high blood pressure, obesity, and peripheral arterial disease have all been recognized as important contributors to the development of AAA, the precise triggers that initiate AAA development remain poorly understood [1,2]. The development of AAA involves multiple pathological processes, including inflammatory responses, oxidative damage, depletion of vascular smooth muscle cells (VSMCs), breakdown of elastic fibers—along with thrombus formation and the emergence of new blood vessels [3]. Given the current lack of targeted pharmacological treatments, exploring previously unrecognized mechanisms involved in AAA progression is essential for uncovering innovative targets that may aid in its diagnosis and therapy [4].

Cellular senescence refers to a biological process in which cells enter a persistent state of growth arrest coupled with sustained metabolic activity, typically triggered by various endogenous and exogenous stressors [5,6]. Replicative senescence arises from the limited division potential of human cells, leading to aging after multiple rounds of cell division [7]. In contrast, premature senescence that occurs independently of telomere shortening can be initiated by multiple stress factors [8,9]. Studies have demonstrated that the senescence of VSMCs, cardiomyocytes, and T cells accumulates in pathologies and can either mitigate or exacerbate the onset and progression of cardiovascular diseases [10,11].

Epidemiological studies indicate that the prevalence of AAA is relatively low in individuals under 55 years of age but sharply increases to approximately 2% at the age of 55, with a continuous rise as age advances [12]. These findings indicate that cellular senescence is a key contributor to both the initiation and advancement of AAA. Basic research further corroborates that in AAA patients, VSMCs, leukocytes, and endothelial cells exhibit senescent changes characterized by alterations in cell morphology, function, and telomere shortening [13–16]. Although several senescence-associated molecules (e.g., SIRT1, miR-1204) have been implicated in AAA pathogenesis, clinically translatable diagnostic biomarkers and targeted therapies specifically derived from integrated senescence signatures remain limited [17]. Therefore, identifying senescence-related biomarkers in AAA, as well as exploring the heterogeneity and dynamics of senescence within AAA, is essential. The innovation of our study lies in the integrative analysis of multiple transcriptomic datasets and senescence-associated genes to identify diagnostic biomarkers [18], delineate their relationship with immune cell infiltration, and define AAA subtypes based on senescence signatures, which was subsequently validated in an animal mode, which may offer novel strategies for diagnosing and managing AAA.

## 2. Methods

### 2.1. Data source

**S1 Fig** presents the overall study protocol. Gene sets associated with senescence were retrieved from the MSigDB (**S1 Table**) [18]. The GSE57961 dataset comprises 49 human abdominal aortic specimens along with 10 control from donors [19]. The GSE183464 dataset, published in 2023, consists of 14 human abdominal aortic specimens [20]. Additionally, The GSE237230 dataset supplies single-cell RNA-sequencing profiles of AAA tissues from 4 patients [20]. The transcriptional mRNA datasets used in this study were obtained from the NCBI GEO repository (https://www.ncbi.nlm.nih.gov/geo/). Detailed information on the publicly available datasets is provided in **Table 1**.

**Table 1:** Transcriptome datasets used in this study.

GSE number	Platform	PMID	Samples	Source types	Group
GSE57691	GPL10558	25944698	49 AAA and 10 HC	AAA tissue	Test dataset
GSE183464	GPL20301	38022704	7 AAA and 7 HC	AAA tissue	Validation dataset
GSE237230	GPL24676	37680984	4 AAA	AAA tissue	Validation dataset

GEO, Gene Expression Omnibus; AAA, abdominal aortic aneurysm; HC, healthy control.

### 2.2. Weighted genes co-expression network analysis (WGCNA)

To explore the association between gene expression patterns and clinical traits, WGCNA was applied [21]. Outlier samples were first identified and removed through hierarchical clustering of samples. Data were processed via the goodSamplesGenes to generate a scale-free network. Applying a soft-thresholding power (β) that satisfied scale-free topology criteria, we calculated network adjacency and derived a topological overlap matrix. Hierarchical clustering followed by module detection with DynamicTreeCut produced initial clusters, which were then merged when their eigengene correlation ＞ 0.75. Finally, modules showing a module-trait correlation greater than 0.5 (P < 0.05) were selected as AAA-related gene sets for subsequent investigations.

### 2.3. Identification of genes exhibiting differential expression patterns

We applied the Bioconductor package to annotate gene expression matrix from the array, averaging values when multiple probes mapped to a single gene symbol. Differentially expressed genes (DEGs) were then identified with the Limma package, using cutoffs of adjusted P-value < 0.05 and |log2 fold change| > 1 [22].

### 2.4. Protein-protein interaction (PPI) network construction

The STRING database was employed to investigate possible PPI networks among the overlapping genes [23]. PPI analysis was performed with a minimum interaction score set to 0.4.

### 2.5. Pathway and ontology enrichment assessment

Senescence-associated DEGs were subjected to enrichment analyses against Gene Ontology (GO), Kyoto Encyclopedia of Genes and Genomes (KEGG), and Reactome repositories. The ten pathways with the strongest enrichment (adjusted P-value < 0.05) are highlighted, and all data handling plus graphical representation were carried out using the R package clusterProfiler [24].

### 2.6. Diagnostic biomarker identification

To uncover senescence-associated indicators for AAA, we applied three machine learning approaches—LASSO regression, random forest analysis, and SVM-RFE. For the LASSO regression analysis, conducted using the glmnet R package, the optimal penalty parameter lambda (λ) was selected via 10-fold cross-validation, choosing the value that gave the simplest model within one standard error of the minimum binomial deviance (lambda.1se). For the random forest analysis, implemented with the randomForest package, 1000 trees (ntree = 1000) were grown, and the number of variables tried at each split was set to the default (square root of the total number of variables). The SVM-RFE algorithm was performed using the e1071 package with a linear kernel, and the feature selection process was evaluated based on accuracy through 5-fold cross-validation. Genes consistently highlighted by all three methods were then designated as candidate diagnostic biomarkers.

### 2.7. Construction of ROC curves and diagnostic nomogram

ROC curves were generated to evaluate the diagnostic performance of each candidate biomarker [25]. The AUC along with the 95% confidence interval (CI) was calculated to quantify predictive accuracy. Biomarkers demonstrating AUC values above 0.7 and showing significant differential expression were selected for nomogram development.

### 2.8. Individual Gene-Based Gene Set Enrichment Analysis

GSEA was conducted based on GO enrichment dataset using clusterProfiler R package with a P-value < 0.05 [24].

### 2.9. Immune infiltration analysis

The CIBERSORT R package was used to quantify immune cell proportions in AAA versus control samples [26], followed by Spearman’s correlation to link these immune fractions with the selected biomarkers. In parallel, the estimate R package generated Immune, Stromal, and ESTIMATE scores for each sample, which were compared between groups [26], and their relationships with biomarker expression were likewise evaluated using Spearman correlation.

### 2.10. Single-cell transcriptomic evaluation of AAA

The GSE237230 dataset comprises single-cell mRNA profiles from four AAA patient specimens. Data preprocessing and integration were performed using Seurat and Harmony packages. Cells with unique feature counts (nFeature_RNA) over 2,500 or less than 200 were filtered out. Cells with a percentage of mitochondrial reads (percent.mt) exceeding 10% were also excluded to remove low-quality cells or potential doublets. A total of 1,407 cells were analyzed. Using the Seurat package, the FindAllMarkers function was applied to detect cluster-specific marker genes, selecting those expressed in at least 25% of the cells and had a log2 fold change (log2FC) >0.25.

### 2.11. Consensus clustering analysis

Consensus clustering analysis using the K-means algorithm was performed with the R package ConsensusClusterPlus to classify AAA subtypes [27]. An optimal cluster number of K = 2 was selected based on evaluation criteria. The expression of Senescence-related AAA biomarker genes was then compared between different subgroups.

### 2.12. Mice

Eight-week-old male C57BL/6J mice were obtained from Vital River Laboratory Animal Technology Co., Ltd. (Beijing, China) and housed in the animal care facility at The First Affiliated Hospital of Nanchang University. The mice were maintained under standard laboratory conditions with a 12-hour light/dark cycle at 25°C. The study design received approval from the Ethics Committee of The First Affiliated Hospital of Nanchang University, located in Nanchang, Jiangxi Province. IACUC (202412GR009).

### 2.13. Induction of AAA in mice

AAA induction surgery was performed according to established protocols adapted for mice [28]. Mice were anesthetized with 4% isoflurane and placed on a heated pad to preserve body temperature. Once the animals demonstrated difficulty maintaining a standing position, anesthesia was sustained using isoflurane delivered via a face mask. Adequate anesthetic depth was confirmed by the absence of response to gentle toe pinching. After shaving the abdominal fur, a middle incision (~1.5 cm) was made through skin and muscle. Visceral contents were gently retracted and under a stereomicroscope the infrarenal abdominal aorta was exposed. A piece of sterile gauze soaked with 10 µL of porcine pancreatic elastase (Type I, E1250, Sigma-Aldrich; at a concentration of 0.5–15.0 mg/mL in water) was applied topically to the exterior of the aorta for 45 minutes. Sham-operated controls underwent an identical procedure, except that heat-inactivated elastase (placed in a boiling water bath for 30 minutes) was used instead. To alleviate pain, buprenorphine was administered subcutaneously every 12 hours for 48 hours postoperatively.

### 2.14. Assessment of aortic expansion

Mice were humanely euthanized through intraperitoneal administration of a lethal dose of sodium pentobarbital. Following euthanasia, the abdominal aorta was carefully dissected free from adjacent connective tissues and imaged ex vivo. For quantification of aneurysm development, the maximum external diameter of the infrarenal aortic segment was determined using ImageJ software (NIH, USA), representing the adventitial width at the region of greatest vascular expansion.

### 2.15. Immunohistochemical analysis

Two weeks post–AAA induction, mice were sacrificed and aortas collected. Sections were then deparaffinized in xylene, rehydrated through graded ethanol series, and subjected to immunostaining with anti-ETS1 antibody (12118-1-AP; Proteintech) and anti-TDO2 antibody (15880-1-AP; Proteintech). Stained slides were quantified with ImageJ (NIH, USA).

### 2.16. Total RNA extraction and qRT-PCR

Peripheral blood was collected from the retro-orbital plexus of mice into EDTA-containing tubes under isoflurane anesthesia. Plasma was separated by centrifugation at 2000 × g for 15 minutes at 4°C for subsequent RNA extraction. Total RNA was isolated from mouse aortic tissue and blood using Extraction Reagent (Vazyme, R401-01). First-strand cDNA was synthesized with Transcription Kit (Vazyme, TR102-01/02). Quantitative PCR was then carried out on the resulting cDNA using SYBR qPCR Mix (Vazyme, Q711) under the following cycling conditions: (1) 95 °C, 3 min; (2) 40 cycles of 95 °C for 3 s and 60 °C for 30 s; (3) Melting curve analysis from 65 °C to 95 °C in 0.5 °C increments. Transcript levels were normalized to GAPDH, and each reaction was performed in triplicate. Primer sequences are listed below. IL6: forward 5’- ACTCACCTCTTCAGAACGAATTG, reverse 5’- CCATCTTTGGAAGGTTCAGGTTG; ETS1: forward 5’- GATAGTTGTGATCGCCTCACC, reverse 5’- GTCCTCTGAGTCGAAGCTGTC; TDO2: forward 5’- AAGGTTGTTTCTCGGATGCAC, reverse 5’- TGTCATCGTCTCCAGAATGGAA; TBX2: forward 5’- CCCCTTCAAGGTGCGAGTC, reverse 5’- TCAGCGGCTACAATGTCCATC; GAPDH: forward 5’- GGAGCGAGATCCCTCCAAAAT, reverse 5’- GGCTGTTGTCATACTTCTCATGG.

### 2.17. Statistical analysis

Differences in DEG and immune cells were assessed by the Kruskal–Wallis’ test. To examine associations between biomarkers and immune cells, spearman’s correlation was applied. For murine experiments, biomarker expression comparisons utilized Student’s t-test. All statistical computations were conducted with GraphPad Prism (v9.1.0) and R (v4.2.3), with significance set at P < 0.05.

## 3. Results

### 3.1. WGCNA of AAA related gene modules

**Fig 1:**
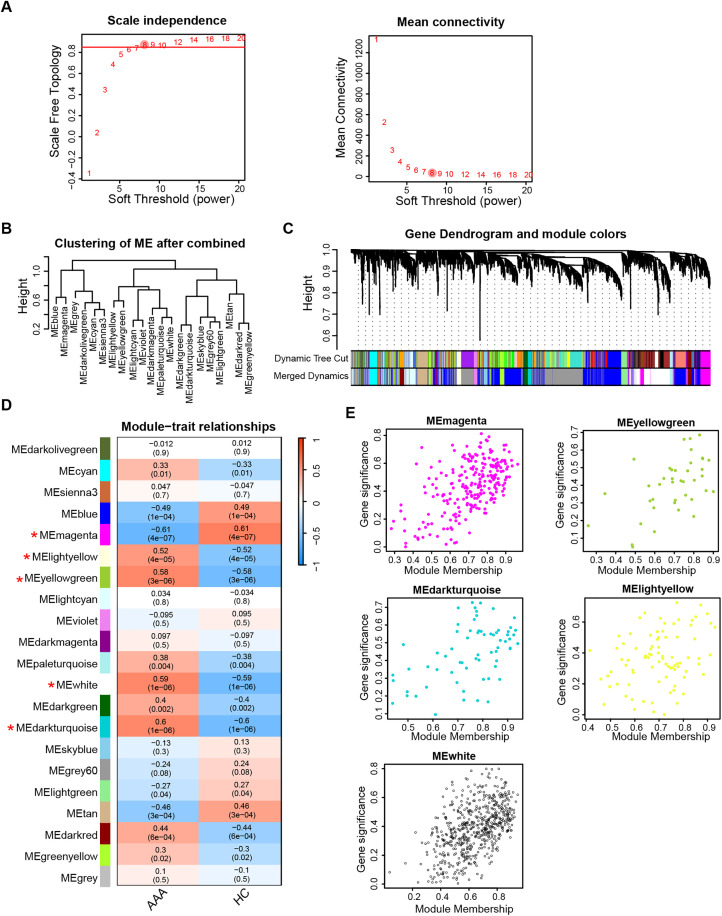
Identification of gene modules associated with AAA. **(A)** Selection of soft threshold power (β) based on scale-free topology model fit and mean connectivity. **(B)** Hierarchical clustering of gene modules post-merging. **(C)** Visualization of the gene dendrogram and module colors derived from hierarchical clustering. **(D)** Heatmap displaying the relationship between gene modules and AAA traits, with gene modules showing a correlation coefficient > 0.5 or <−0.5 and a P-value < 0.05 marked by an asterisk (*). **(E)** Correlation analysis between module membership and gene significance within the key gene modules. AAA, abdominal aortic aneurysm; HC, healthy control.

Initially, we detect critical gene modules associated with AAA. As shown in **Fig 1A**, we determined the optimal soft-threshold power to be 8 based on model fit and mean connection. Then hierarchical clustering led to the identification of distinct co-expression modules, each represented by different colors (**Fig 1B and 1C**). The correlation between AAA and gene modules is depicted in **Fig 1D**, with corresponding genes listed in **S2 Table**. Notably, gene modules showing a coefficient greater than 0.5 or less than –0.5, along with a P-value below 0.05, were considered key modules and selected for downstream analysis (**Fig 1E**).

### 3.2. DEGs between AAA and control

**Fig 2:**
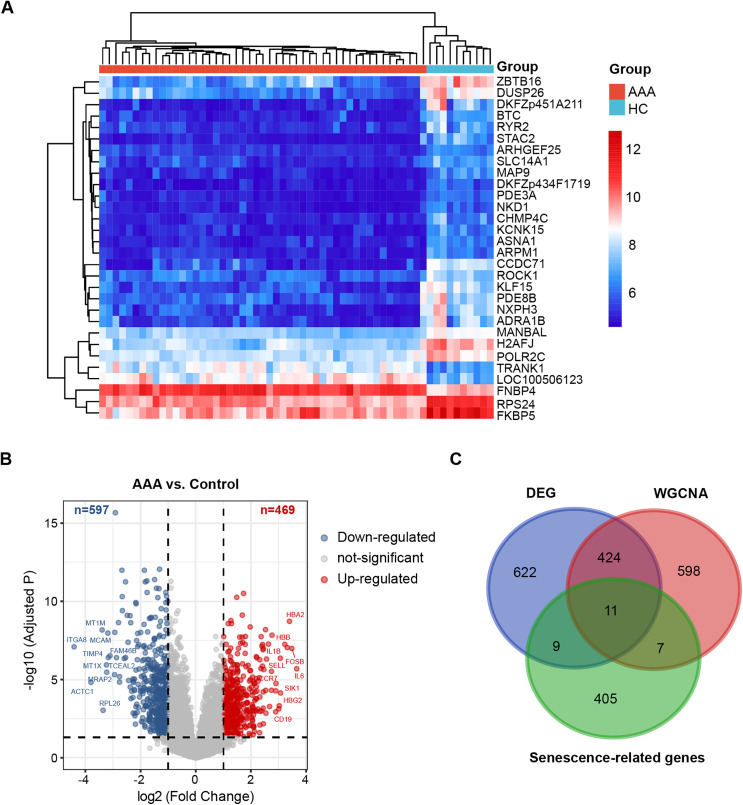
Identification of DEGs between AAA and control groups. **(A)** Heatmap showcasing the top 30 most significant DEGs. **(B)** Volcano plot illustrating DEGs between AAA and control groups, with red circles indicating up-regulated genes (log2 FC > 1, adjusted P < 0.05) and blue circles indicating down-regulated genes (log2 FC < −0.5, adjusted P < 0.05). **(C)** Identification of overlapping genes among WGCNA-derived key module genes, DEGs, and senescence-related genes. WGCNA, weighted genes co-expression network analysis; DEGs, differentially expressed genes.

As shown in **Fig 2A**, the top 30 DEGs with the highest statistical significance are highlighted. In total, 1,066 DEGs were detected, comprising 469 upregulated genes (P < 0.05, log2FC > 1) and 597 downregulated genes (P < 0.05, log2FC < –1) (**Fig 2B**). The full list of DEGs is available in **S2** **and** **S3 Tables**. Following the intersection with senescence-related genes, 11 overlapping genes were defined as senescence-associated DEGs in AAA (**Fig 2C**).

### 3.3. Functional analysis of senescence-related DEGs

**Fig 3:**
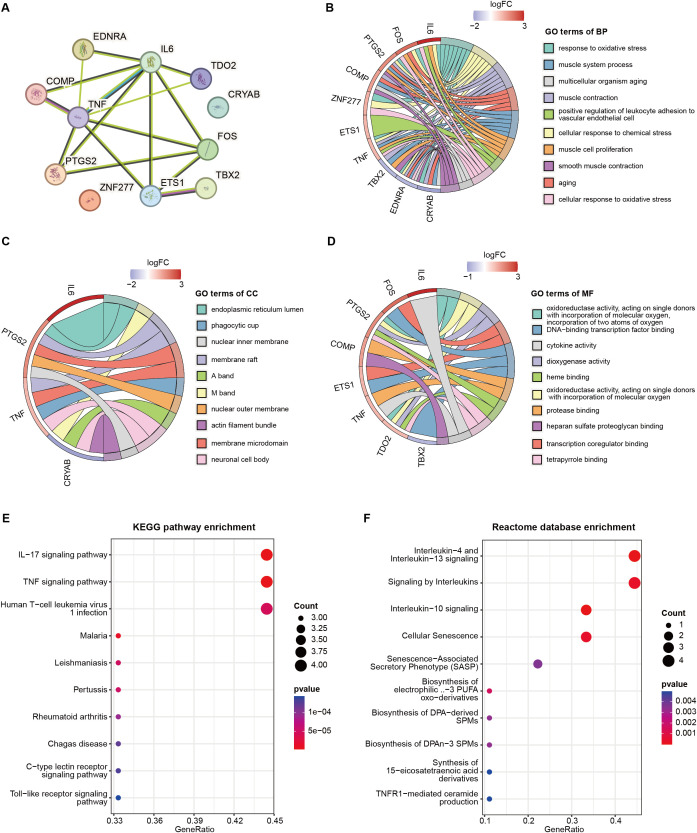
Functional analysis of senescence-related DEGs in AAA. **(A)** PPI network illustrating the potential interactions among 11 senescence-related DEGs in AAA. (**B)**, **(C)**, and (**D**) display the top 10 most significant enrichment GO terms for Biological Process (BP), Cellular Component (CC), and Molecular Function (MF) of these 11 senescence-related DEGs. (**E**) and (**F**) present the top 10 most significant pathways enriched in KEGG and REACTOME, respectively.

To uncover the potential interplay of the 11 senescence-related DEGs, we we generated a PPI network of 11 nodes and 16 edges using STRING database (**Fig 3A**). The top 10 most enriched GO terms for biological processes (BP) (**Fig 3B**), cellular components (CC) (**Fig 3C**), and molecular functions (MF) (**Fig 3D**) were displayed, revealing associations with oxidative stress, leukocyte migration and activation, and smooth muscle cell function. KEGG pathway analysis identified cytokine activation, including IL-17 and tumor necrosis factor (TNF), as significantly enriched (**Fig 3E**). Similarly, Reactome pathway analysis highlighted the impact of immune and inflammatory responses on senescence-related AAA (**Fig 3F**). Comprehensive results of the functional enrichment analysis can be found in **S4****–****S6 Tables**.

### 3.4. Candidate AAA biomarkers related to senescence

**Fig 4:**
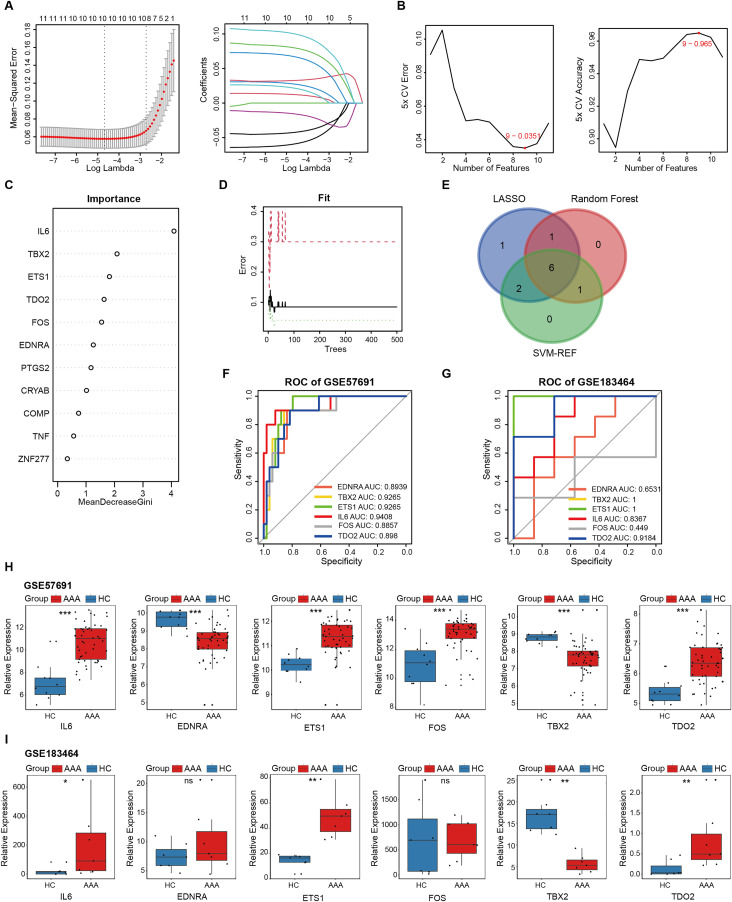
Recognition of senescence-related biomarkers in AAA. **(A)** LASSO logistic regression was utilized to identify senescence-related DEGs with the lowest binomial deviance. **(B)** Hub genes were selected based on the highest accuracy and lowest error after 5-fold cross-validation using the SVM-RFE algorithm. **(C)** Senescence-related DEGs were ranked by importance through random forest analysis. **(D)** The diagnostic error for HC, AAA, and total groups was visualized using random forest. **(E)** Venn diagram illustrating the overlap of 6 biomarkers identified by the three algorithms. ROC curves were generated from the 6 biomarkers in GSE57691 (**F**) and GSE183464 **(G)**. Comparison of the normalized expression levels of the 6 biomarkers in GSE57691 (**H**) and GSE183464 **(I)**. Kruskal-Walli’s test was applied in (**H**) and **(I)**. *, P < 0.05; **, P < 0.01; ***, P < 0.001; ns, not significant.

Firstly, LASSO regression analysis was conducted to select the 10 genes exhibiting the lowest deviance, indicating their strong predictive value, as shown in **Fig 4A**. Using the SVM-RFE algorithm with 5-fold cross-validation, 9 genes were selected as the most robust candidates (**Fig 4B**). Additionally, random forest analysis was used to evaluate the importance of the 11 senescence-related genes, ultimately identifying 8 genes with the highest predictive relevance (**Fig 4C and 4D**). The complete list of genes selected by these machine learning algorithms is provided in **S7 Table**. Following intersection, IL6, EDNRA, ETS1, FOS, TBX2 and TDO2 were identified as potential biomarkers based on their consistent selection across multiple analytical methods (**Fig 4E**). The AUCs values for these biomarkers were computed (**Fig 4F and 4G**). Additionally, their expression levels were compared in both the training dataset (**Fig 4H**) and the validation dataset (**Fig 4I**). Following stringent evaluation, IL6, ETS1, TDO2, and TBX2 selected as final biomarkers due to their robust diagnostic performance (AUC > 0.7) and statistically significant expression.

### 3.5. Establishment of nomogram and GSEA

To evaluate the diagnostic strength of the senescence-associated biomarkers, we created a nomogram incorporating the expression levels of IL6, ETS1, TDO2, and TBX2 (**S2A Fig**). The ROC curve of the nomogram demonstrated its strong diagnostic value, with an AUC of 1 (**S2B Fig**). It should be noted that this result, likely influenced by the limited sample size, must be interpreted with caution as it suggests a risk of model overfitting. The generalizability of this model requires validation in larger, independent cohorts.

Single-gene GSEA based on the GO database further elucidated the potential pathways associated with these biomarkers. GSEA for IL6 indicated its primary involvement in oxidative stress and apoptosis pathways (**S2C Fig**). For ETS1 and TDO2, GSEA revealed their significant roles in ribosome assembly, endoplasmic reticulum stress, and cell membrane localization (**S2D** **and** **S2E Fig**). GSEA for TBX2 suggested its involvement in adaptive immune response processes during AAA development. Detailed results of the top 5 most significant pathways from the GO-based single-gene GSEA are provided in **S8 Table**.

### 3.6. Immune infiltration analysis of AAA

**Fig 5:**
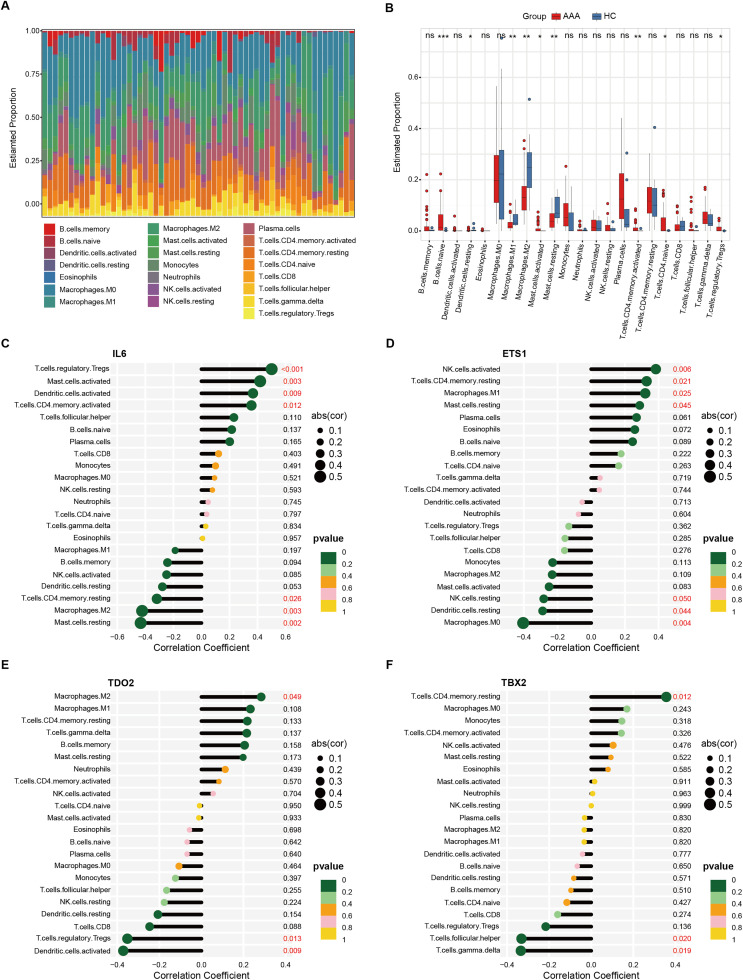
Immune infiltration landscape of AAA. **(A)** Bar plot depicting the relative proportions of immune cell types infiltrated in AAA compared to controls. **(B)** Differential proportions of immune cell populations between AAA and control groups. The correlation between immune cell types and the expression levels of IL6 **(C)**, ETS1 **(D)**, TDO2 **(E)**, and TBX2 **(F)** in AAA. Kruskal-Walli’s test in **(B)**, Spearman correlation analysis in **(C)**, **(D)**, **(E)**, and **(F)**. *, P < 0.05; **, P < 0.01; ***, P < 0.001; ns, not significant.

To investigate the association between immune infiltration and identified biomarkers, we performed an immune infiltration analysis. The relative proportions of various immune cell types are depicted in the bar plot (**Fig 5A**). Notable differences in immune cell composition were observed between AAA and control (**Fig 5B**). Further correlation analysis revealed a significant association between senescence-related biomarkers and the relative proportions of various immune cell populations (Fig 5C**–5F**).

Given the significant role of immune inflammation in AAA, we subsequently applied the ESTIMATE algorithm to quantify the immune and stromal components within the AAA microenvironment. The results demonstrated that stromal scores (**S3A Fig**), immune scores (**S3B Fig**) and ESTIMATE scores (**S3C Fig**) were elevated compared to control. Correlation analysis further confirmed the close relationship between senescence-related biomarkers and these immune scores, with IL6, ETS1, and TDO2 exhibiting positive correlations, while TBX2 showed a negative correlation (**S3D Fig**).

### 3.7. Single-cell mRNA-seq of AAA

**Fig 6:**
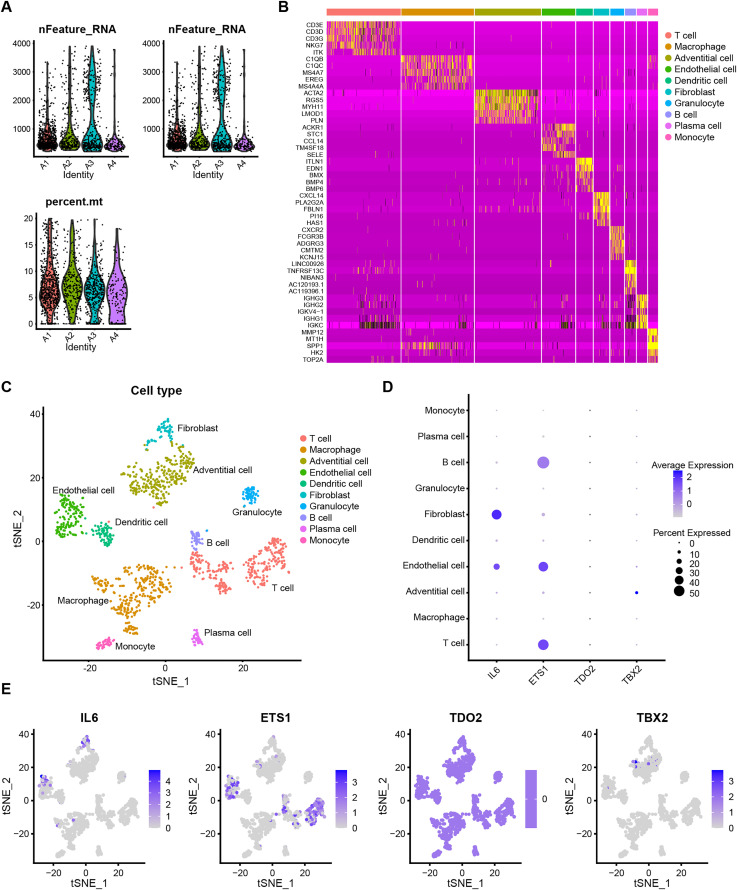
Single-cell RNA-seq analysis of AAA. **(A)** Violin plot showing the distribution of single-cell sequencing data for each AAA sample after quality control. **(B)** Heatmap displaying the expression of characteristic genes across single-cell subpopulations in AAA tissue. **(C)** t-SNE plot illustrating the presence of 10 distinct immune cell types within AAA tissue. **(D)** Dot plot and **(E)** feather plot presenting the expression patterns of IL6, ETS1, TDO2, and TBX2 across various cell populations.

To better visualize the expression profiles of senescence-associated biomarkers in AAA tissues, we conducted a detailed analysis of single-cell mRNA sequencing data derived from four AAA patient samples, yielding a total of 1,407 cells. **Fig 6A** illustrate the distribution of cells and genes across different samples. Using t-SNE dimensionality reduction, we identified ten distinct cell populations and depicted the expression patterns of corresponding marker genes within these populations (**Fig 6B and 6C**). The ten distinct cell populations were annotated based on the expression of well-established canonical marker genes. Detailed annotations of the marker genes are provided in **S9 Table**. **Fig 6D and 6E** show the relative expression levels of senescence-related biomarkers across different cell subtypes. Specifically, IL6 is mainly expressed in endothelial cells, fibroblasts, and macrophages, whereas ETS1 shows expression in endothelial cells, T cells, and B cells. TDO2 exhibits consistent expression across all cell types, whereas TBX2 is predominantly expressed in adventitial cells. The expression patterns of IL6 in macrophages/fibroblasts and ETS1 in endothelial/T cells suggest potential paracrine signaling axes (e.g., macrophage-derived IL-6 influencing vascular cell senescence) within the AAA microenvironment.

### 3.8. Subtypes identification of AAA

To explore potential subtypes within AAA and the relationship with senescence-related biomarkers, we conducted a consensus clustering analysis of AAA samples. Using K-means clustering with the number of clusters (K) varying from 2 to 6, we generated corresponding consensus matrix heatmaps (**S4A** **and** **S4B Fig**). The robustness of the clustering was assessed using CDF distribution (**S4C Fig**) and the Delta area (**S4D Fig**), indicating that partitioning the samples into two groups, A and B, provided the most stable clustering solution. **S4E Fig** displays the clustering assignments of samples across the various K values tested. PCA analysis further validated the distribution of these two AAA clusters, revealing overall differences in gene expression between the two clusters (**S4F Fig**). Notably, ETS1 and TDO2 were significantly upregulated in cluster A, while IL6 was prominently elevated in cluster B (**S4G Fig**). TBX2 expression showed no statistically significant difference between the two clusters. These findings indicate that the expression patterns of senescence-related genes are key determinants in classifying AAA subtypes.

### 3.9. Murine experiment validation

**Fig 7:**
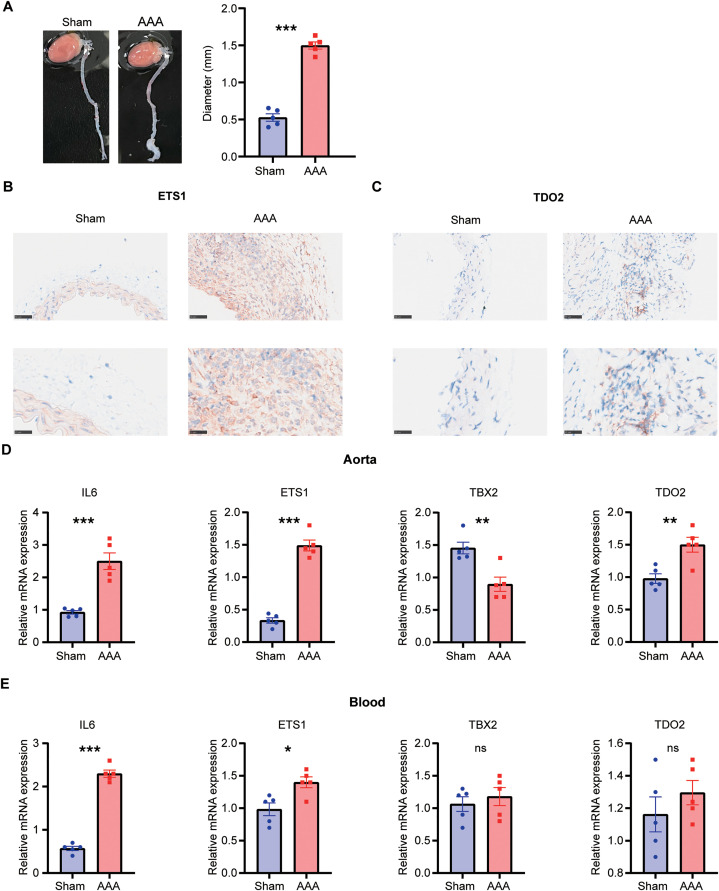
Validation of biomarker expression in a murine AAA model. **(A)** Representative images of the general aorta from sham-operated and AAA groups, with aortic diameter quantified (n = 5). Immunohistochemistry images showing ETS1 **(B)** and TDO2 **(C)** staining in sham-operated versus AAA groups. qPCR analysis of IL6, ETS1, TBX2, and TDO2 expression levels in murine aortas **(D)** and blood **(E)** 14 days after operation (n = 5). Students’ t test. *, P < 0.05; **, P < 0.01; ***, P < 0.001; ns, not significant.

To confirm the altered expression of senescence-associated biomarkers in AAA, we established a murine AAA model (**Fig 7A**). Immunohistochemical analysis demonstrated markedly elevated ETS1 and TDO2 expression levels in AAA tissues (**Fig 7B and 7C**). Consistent with our observations from public databases, we observed elevated expression of IL6, ETS1, and TBX2 in AAA, while TDO2 expression was reduced (**Fig 7D**). Additionally, expression profiles of biomarkers in peripheral blood samples cells closely mirrored those observed in AAA tissues (**Fig 7E**), suggesting a potential link between local immune cells in AAA and peripheral blood immune cells.

## 4. Discussion

As modern society advances, the issue of population aging has become increasingly prominent. AAA, as a senescence-related disease, exhibits a marked rise in incidence with advancing age [12]. Epidemiological studies show that among men aged 65–74, the incidence of AAA is 55 cases per 100,000 person-years, rising to 112 cases in those aged 75–85, and sharply increasing to 298 cases per 100,000 person-years in men older than 85 [29]. AAA is a progressive cardiovascular disorder marked by the apoptosis of VSMCs and infiltration of immune cells, closely aligning with hallmark features of cellular senescence, such as reduced proliferative capacity and stress-induced premature senescence [17,30]. Moreover, studies revealed that the telomere length of vascular endothelial cells and leukocytes appeared significantly shortened in patients with AAA [14,31].

Senescence is a critical risk factor for a variety of vascular diseases, including AAA and aortic dissection [32]. First, it induces endothelial cell dysfunction, impairing their ability to respond dynamically to vasorelaxation and vasoconstriction stimuli, thus predisposing them to a pro-thrombotic state [33]. Second, senescence stimulates VSMCs to secrete inflammatory mediators and drives extracellular matrix remodeling, which together contribute to heightened vascular stiffness [34,35]. Third, it enhances the infiltration of immune cells and promotes the sustained release of pro-inflammatory mediators during the progression of the disease. [36]. Given the lack of effective pharmacological treatments for AAA, with surgical intervention being the primary therapeutic option, investigating senescence-related mechanisms in AAA presents a promising avenue for developing novel diagnostic and therapeutic strategies [4].

By intersecting the module genes, DEGs, and senescence-related genes associated with AAA, we identified 11 senescence-related DEGs specific to AAA. Enrichment analysis suggested key pathways related to AAA pathogenesis, including inflammatory responses, VSMCs dysfunction, and oxidative stress. Persistent inflammation—a defining feature of cellular senescence—is tightly associated with the senescence process itself [6]. Inflammation refers to the state where the immune system fails to clear senescent cells and pro-inflammatory factors, leading to elevated inflammation [6,37]. This ongoing, low-level inflammatory state gradually intensifies with age and contributes to the aging process, creating a self-perpetuating vicious cycle [38]. Our results suggest that cytokines like IL-17 and TNF-α may serve as promising targets within chronic inflammatory pathways linked to senescence-driven AAA. VSMCs are essential for preserving vascular development and homeostasis [39]. However, when exposed to senescence-associated stressors, VSMCs transition from a contractile to a synthetic phenotype, leading to the secretion of harmful mediators such as ROS, pro-inflammatory cytokines, and MMPs. [40]. This is accompanied by impaired proliferation and increased apoptosis, suggesting that the senescence-related phenotypic transformation of VSMCs plays a pivotal role in the progression of AAA [41].

To investigate senescence-related biomarkers of AAA, we employed machine learning algorithms and identified four key genes: IL-6, ETS1, TDO2, and TBX2. IL-6, a pleiotropic pro-inflammatory cytokine, plays a crucial role in initiating acute phase responses [42]. Previous research has shown that increased serum IL-6 levels are positively associated with aneurysm size in patients with AAA [43,44]. Yuwen et al. also reported increased IL-6 expression in AAA, while treated with IL-6 antagonism preventing aneurysm progression and rupture [45]. Additionally, senescence elevates circulating IL-6, promoting maladaptive clonal hematopoiesis in the bone marrow, leading to increased inflammatory infiltration into the diseased aorta [46,47]. ETS1, a member of the ETS transcription factor family, serves as a crucial regulator of both B and T lymphocyte function [48]. Genetic variants in the human ETS1 gene have been associated with an increased risk of developing autoimmune and inflammatory disorders such as rheumatoid arthritis (RA), psoriasis, and ankylosing spondylitis [49–51]. Notably, patients with these conditions exhibit reduced ETS1 mRNA levels in immune cells [48]. However, our study revealed elevated ETS1 expression in AAA tissues, corroborating findings from other research that implicate ETS1 in promoting angiogenesis and endothelial apoptosis, processes mirrored in AAA pathology. Furthermore, ETS1 has been shown to induce cellular senescence by downregulating ribosomal activity, suggesting its potential pathogenic role in AAA [52].

TDO2 defined as a heme-containing dioxygenase, which converting tryptophan into kynurenic acid [53]. Studies have shown that kynurenine pathway intermediates, including kynurenine, play a critical part in activating immune and inflammatory cells [54]. In addition, TDO2 is found to be expressed across multiple cancer types [55], which facilitates tumor immune escape and impairs T cell function [56]. Research also shows that elevated TDO2 expression increases levels of inflammatory cytokines such as IL-6 and IL-1β [57]. In our study, we observed increased TDO2 expression in AAA tissues compared to controls, suggesting that TDO2 may play a role in mediating inflammation in AAA. TBX2, a member of the T-box transcription factor family, primarily acts as a transcriptional repressor [58,59]. Additionally, TBX2 overexpression in uterine myometrial cells has been shown to downregulate TNFα and interferon signaling, suppressing inflammatory pathways [60]. Consistent with these findings, our research indicated a downregulation of TBX2 in AAA tissues, which may exacerbate inflammation. However, the precise function of TBX2 in the development and progression of AAA remains to be fully elucidated.

Given the critical role of inflammation in AAA, we conducted further analyses of the immune microenvironment and senescence-related biomarkers. The results indicate that IL-6 is predominantly expressed in endothelial cells and macrophages, whereas ETS1 expression is mainly found in endothelial cells and lymphocytes. TDO2 exhibited uniform expression across all cell types, whereas TBX2 was predominantly expressed in aortic adventitial cells. Single-gene GSEA revealed that ETS1 and TDO2 were associated with ribosomal activity and protein membrane localization, and TBX2 was implicated in adaptive immune regulation. These findings highlight the potential roles of these markers and the specific cell types in AAA. In addition, Estimate scores were elevated in AAA tissues, indicating a more severe immune inflammatory infiltration and extracellular matrix formation in AAA. Correlation analysis suggests that TBX2 may function as an anti-inflammatory gene, providing a promising target for future therapeutic investigations in AAA.

Clinical research has demonstrated that AAAs with larger diameters have a higher risk of rupture and hemorrhage, leading to the categorization of aneurysms into stable and unstable subtypes according to their size [61]. To investigate potential disease subtypes within AAA, we employed consensus clustering and categorized all AAA patients into two subgroups, A and B. Notably, the expression of senescence-related biomarkers IL6, ETS1, and TDO2 differed significantly between these two subgroups, suggesting that senescence-related gene expression may serve as a key indicator for AAA subtyping. While cellular senescence is a well-established factor in AAA, most prior studies have focused on individual genes or pathways. Our study provides added value through a triangulated, multi-method approach. Furthermore, a key translational insight from our work is the use of this senescence signature to stratify AAA patients into two distinct molecular subtypes. This subtyping suggests that senescence heterogeneity may underlie clinical variability, moving beyond gene discovery towards potential patient stratification. This finding aligns with clinical research linking senescence to increased AAA diameter and rupture rates, further underscoring role of senescence in AAA.

Our study provides the first systematic investigation of the role of senescence as a pathological process in the progression of AAA, utilizing public transcriptomic datasets and senescence-related gene. We identified 11 senescence-associated DEGs in AAA. Enrichment analysis indicated that they mainly associated with oxidative stress pathways, immune-inflammatory responses, and dysfunction of VSMCs. Using three machine learning approaches, we selected IL6, ETS1, TDO2, and TBX2 as biomarkers related to senescence in AAA, with nomogram demonstrating their strong predictive value for AAA diagnosis. Immune infiltration and single-cell analyses highlighted a robust connection between genes and immune-inflammatory cells. Consensus clustering analysis identified two subtypes of AAA and revealed significant expression differences of aging-related biomarkers between these subtypes. Finally, we confirmed the altered expression of these biomarkers using a murine model of AAA.

While abdominal aortic aneurysm is ultimately diagnosed through structural imaging, significant challenges persist in early detection, risk stratification, and targeted intervention. Our study establishes that cellular senescence plays a central role in AAA pathogenesis, providing new perspectives on these clinical dilemmas. The four-gene senescence signature (IL-6, ETS1, TDO2, TBX2) demonstrates robust diagnostic capability, suggesting its potential utility as supplementary biomarkers for early AAA detection in high-risk populations. Furthermore, the identification of two distinct AAA subtypes based on senescence signatures addresses the critical need for better risk stratification. This molecular subtyping approach could eventually guide personalized surveillance strategies and help identify patient subgroups that might benefit from targeted interventions against specific senescence-associated pathways. While further validation in prospective cohorts is needed, our findings provide a framework for developing more precise approaches to AAA management that address the current limitations of relying solely on anatomical measurements.

Our study may offer new insights and targets for AAA. Nevertheless, our study has certain limitations. First, the sample size of the primary transcriptomic dataset (GSE57691) is modest. The perfect diagnostic performance (AUC = 1.0) of our nomogram in the training set is a strong indicator of overfitting, and the generalizability of this four-gene signature must be rigorously validated in future large-scale, prospective cohorts before any clinical application can be considered. Second, while we validated expression changes in a murine model, our study lacks functional validation (e.g., knockout or overexpression) to establish a direct causal link between IL6, ETS1, TDO2, TBX2 and senescence or inflammatory phenotypes in AAA. Thus, these genes are best regarded as promising candidate biomarkers for further investigation. Besides, a limitation of our subtyping analysis is the lack of detailed clinical data (e.g., age, gender, aneurysm growth rate, or rupture events) in the public datasets used. Therefore, we could not directly correlate the identified subtypes with clinical features or prognosis. Future studies with well-annotated, prospective clinical cohorts are essential to validate whether this senescence-based subtyping strategy can predict disease progression, rupture risk, or response to potential therapies. Third, although we performed single-cell analysis to map gene expression, the available data did not allow us to directly colocalize our signature with canonical senescence markers at the single-cell level, nor to perform detailed intercellular communication analysis. Future studies employing targeted assays or spatial transcriptomics are needed to fully elucidate these interactive networks.

## 5. Conclusion

Our study demonstrates that senescence-associated genes are pivotal in AAA development by modulating ROS, immune-mediated inflammation, and dysfunction of VSMCs. We identified IL6, ETS1, TDO2, and TBX2 as a candidate biomarker signature associated with AAA diagnosis and elucidated their expression patterns and potential functions. Furthermore, our analysis of AAA subtypes revealed significant expression differences of senescence-related biomarkers. Finally, we confirmed the altered expression of these biomarkers in a murine AAA model. These results may provide a foundational framework for future research of AAA.

## Supporting information

S1 Fig The flow work of the study. (DOCX)

S2 Fig Diagnostic nomogram for AAA and single-gene GSEA. (DOCX)

S3 Fig Analysis of the immune microenvironment in AAA. (DOCX)

S4 Fig Exploration of potential subtypes in AAA using consensus clustering approach. (DOCX)

S1 Table List of senescence-related genes. (XLSX)

S2 Table WGCNA results of module colors to genes. (XLSX)

S3 Table Full list of DEGs between AAA and control. (XLSX)

S4 Table Top 10 most significant GO enrichment results of senes-cence-related DEGs. (XLSX)

S5 Table Top 10 most significant KEGG enrichment results of se-nescence-related DEGs. (XLSX)

S6 Table Top 10 most significant Reactome enrichment results of senescence-related DEGs. (XLSX)

S7 Table Candidate biomarkers identified by machine learning al-gorithms from senescence-related DEGs. (XLSX)

S8 Table Single-gene GSEA of the biomarkers based on GO database. (XLSX)

S9 Table Marker genes used for annotating cell types of the ten immune cell clusters. (XLSX)
