# Supplementary material for: Elucidate senescence-related gene signature and immune infiltration landscape in abdominal aortic aneurysm
Source: PLoS One. 2026 Jan 20;21(1):e0340976. doi: 10.1371/journal.pone.0340976 (PMC12818648; doi:10.1371/journal.pone.0340976)
Supplement: S1 Fig — (DOCX) [file pone.0340976.s001.docx]

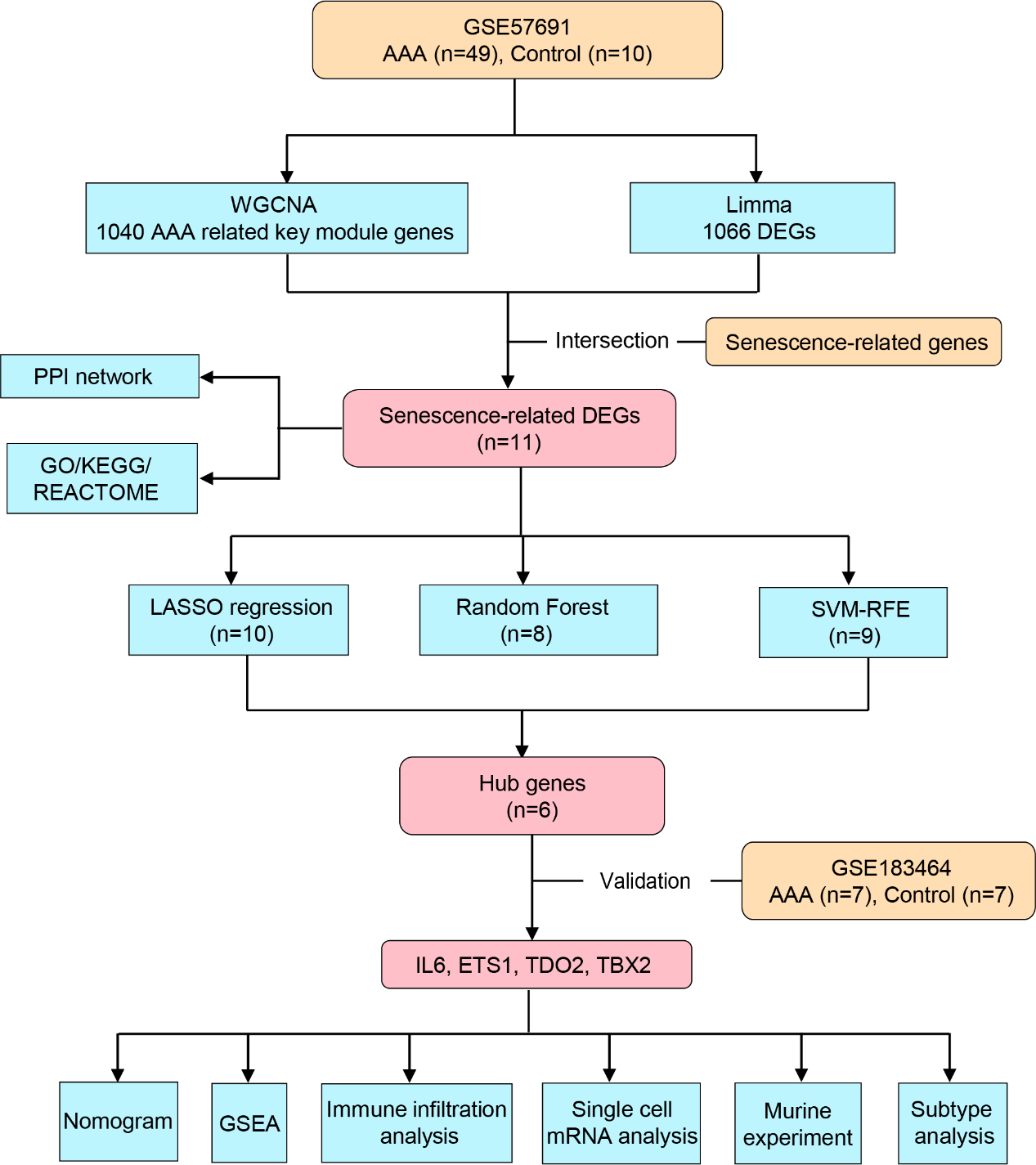


**Supplementary Figure S1. The flow work of the study.** AAA, abdominal aortic aneurysm; WGCNA, weighted genes co-expression network analysis; DEGs, differentially expressed genes; PPI, protein-protein interaction; GO, gene ontology; KEGG, Kyoto Encyclopedia of Genes and Genomes; LASSO, least absolute shrinkage and selection operator; SVM-RFE, support vector machine recursive feature elimination; GSEA, Gene set enrichment analysis.
