# Supplementary material for: Elucidate senescence-related gene signature and immune infiltration landscape in abdominal aortic aneurysm
Source: PLoS One. 2026 Jan 20;21(1):e0340976. doi: 10.1371/journal.pone.0340976 (PMC12818648; doi:10.1371/journal.pone.0340976)
Supplement: S2 Fig — (DOCX) [file pone.0340976.s002.docx]

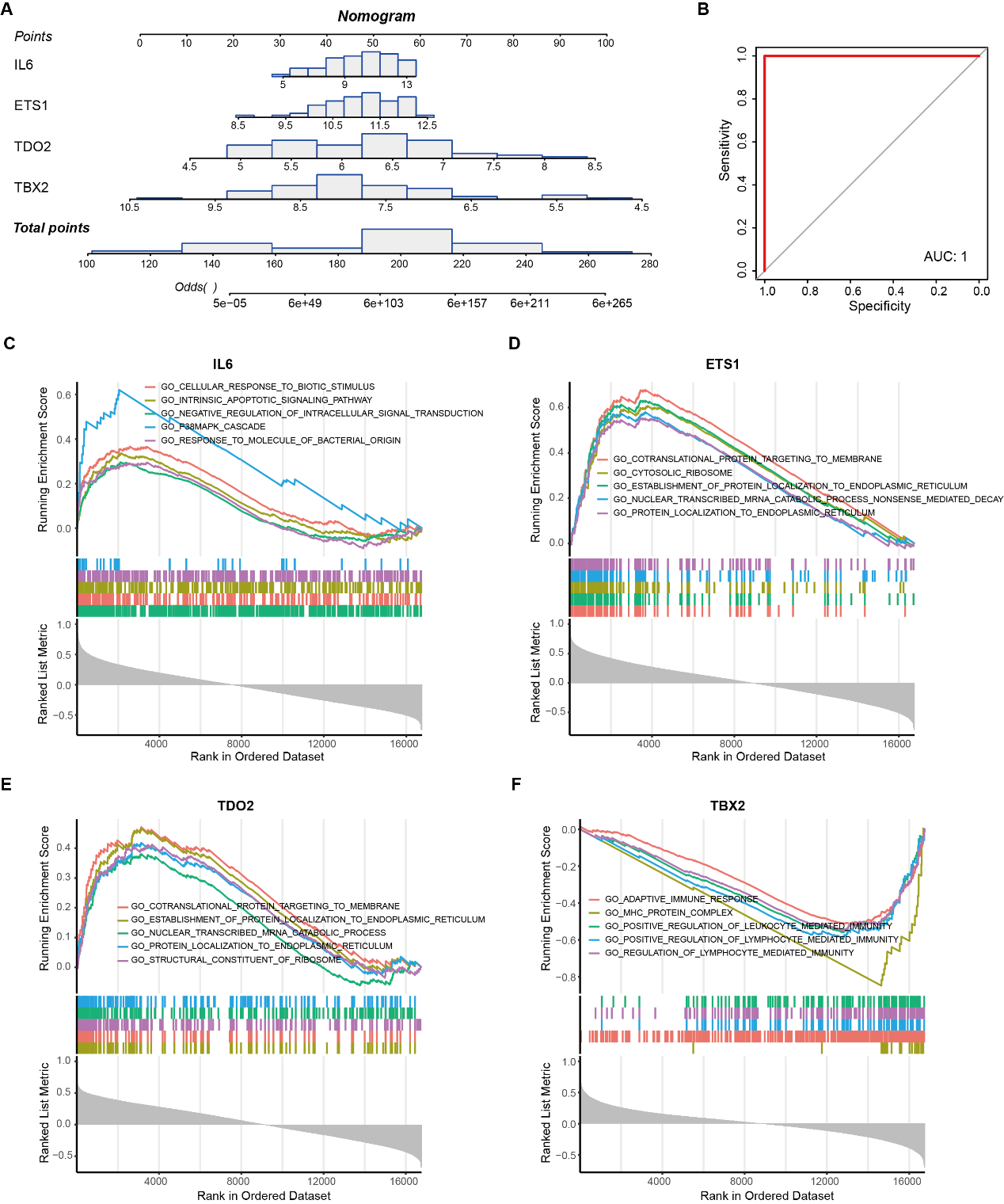


**Supplementary Figure S2. Diagnostic nomogram for AAA and single-gene GSEA. (A)** Nomogram constructed based on the expression of senescence-related biomarkers in AAA. **(B)** ROC curve derived from the diagnostic nomogram, assessing its predictive accuracy. Single-gene GSEA for IL6 **(C)**, ETS1 **(D)**, TDO2 **(E)**, and TBX2 **(F)** highlighting the top 5 most significant GO enrichments associated with each gene.
