# Supplementary material for: Elucidate senescence-related gene signature and immune infiltration landscape in abdominal aortic aneurysm
Source: PLoS One. 2026 Jan 20;21(1):e0340976. doi: 10.1371/journal.pone.0340976 (PMC12818648; doi:10.1371/journal.pone.0340976)
Supplement: S3 Fig — (DOCX) [file pone.0340976.s003.docx]

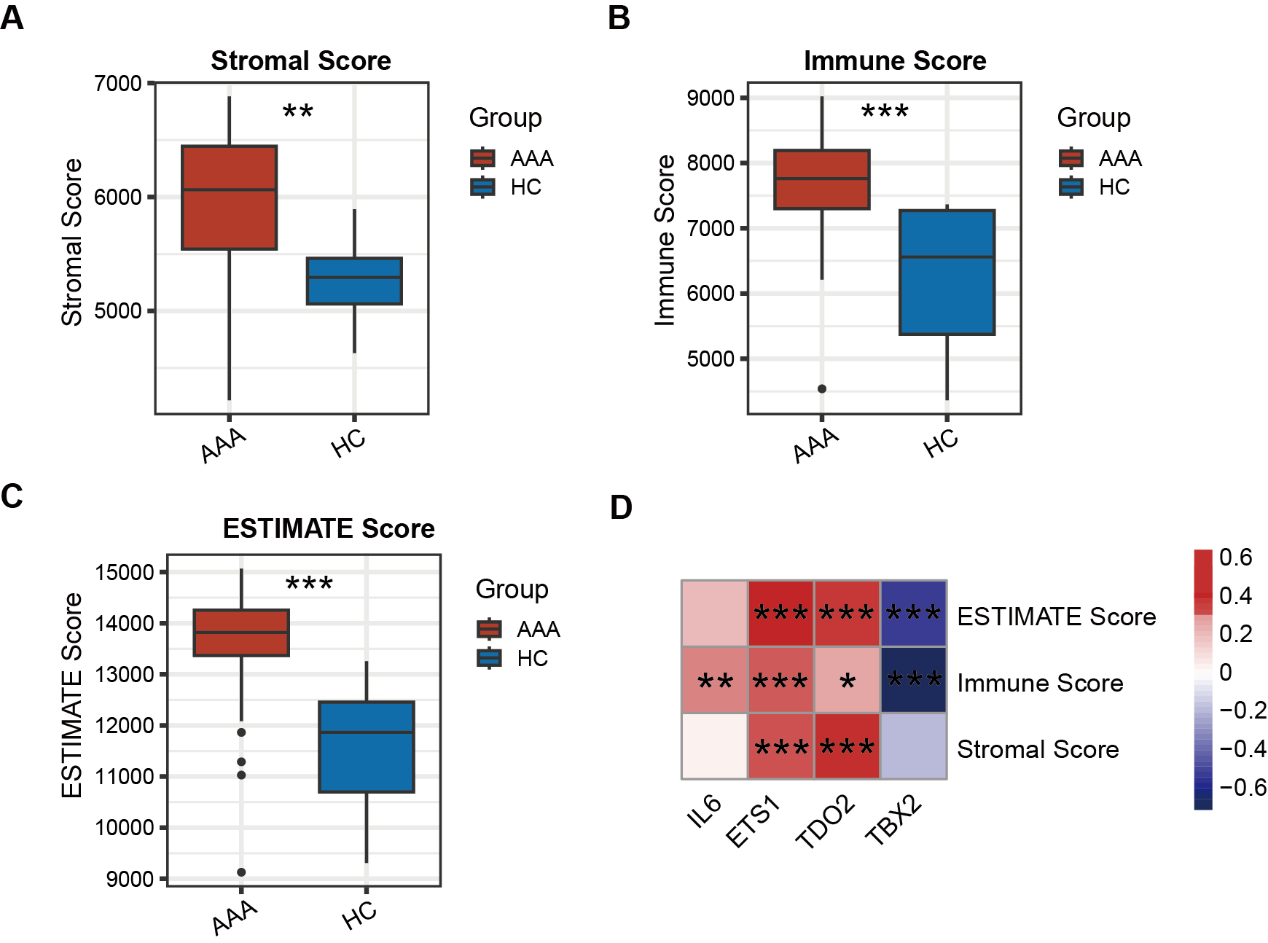


**Supplementary Figure S3. Analysis of the immune microenvironment in AAA.** Comparison of Stromal Score **(A)**, Immune Score **(B)**, and ESTIMATE Score **(C)** between AAA and control samples. **(D)** Correlation analysis of these scores with the expression levels of IL6, ETS1, TDO2, and TBX2. Kruskal-Walli’s test in **(A)**, **(B)**, and **(C)**. Spearman correlation analysis in **(D)**. AAA, abdominal aortic aneurysm; HC, healthy control. *, P<0.05; **, P<0.01; ***, P<0.001; ns, not significant.
