# Supplementary material for: Elucidate senescence-related gene signature and immune infiltration landscape in abdominal aortic aneurysm
Source: PLoS One. 2026 Jan 20;21(1):e0340976. doi: 10.1371/journal.pone.0340976 (PMC12818648; doi:10.1371/journal.pone.0340976)
Supplement: S4 Fig — (DOCX) [file pone.0340976.s004.docx]

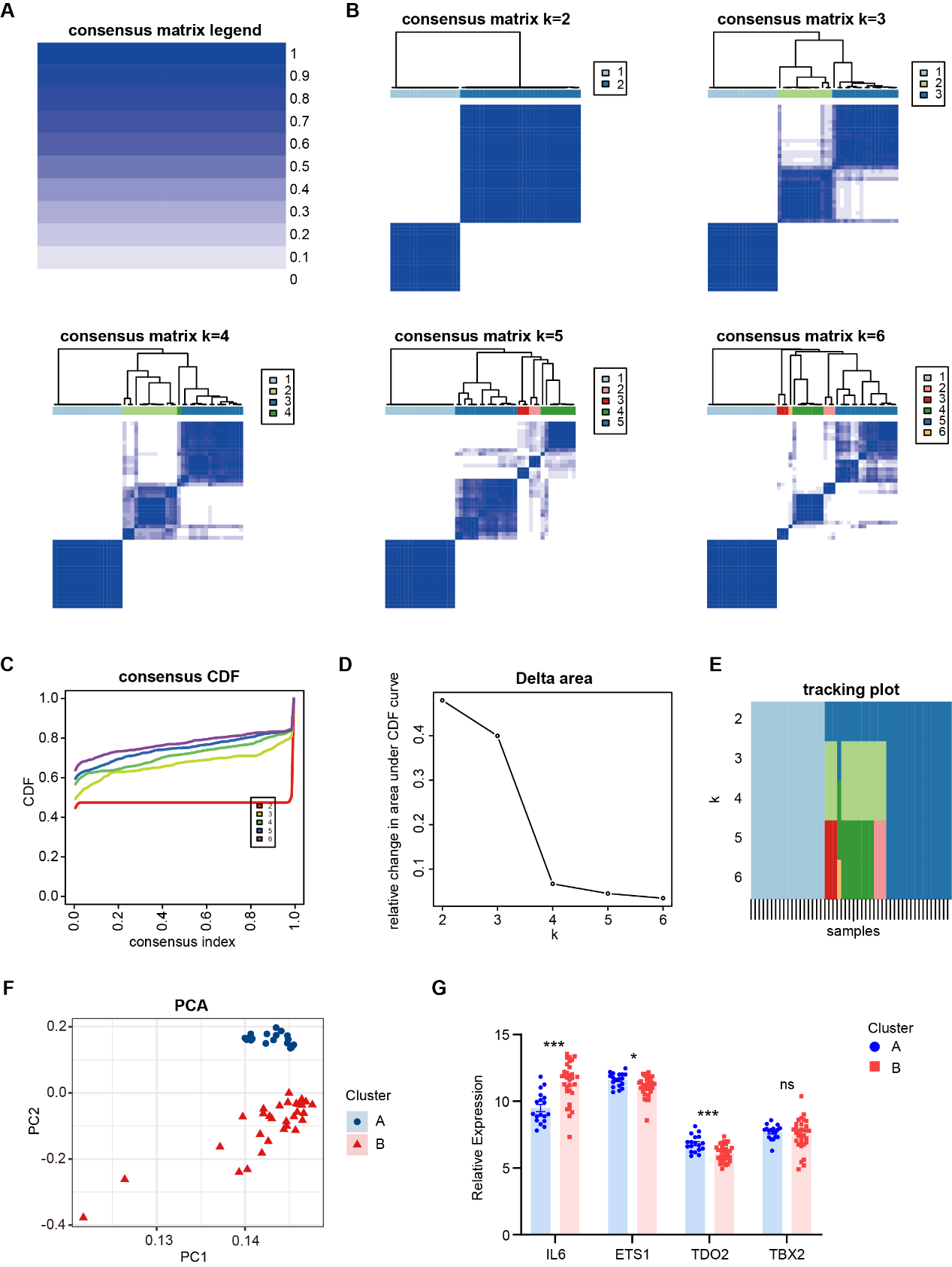


**Supplementary Figure S4. Exploration of potential subtypes in AAA using consensus clustering approach. (A)** Color legend for the heatmap generated from the consistency matrix. **(B)** Heatmaps of consistency matrices for different values of k (k=2-6). **(C)** Cumulative distribution function (CDF) plots for varying numbers of clusters. **(D)** Relative change in the area under the CDF curve. **(E)** Variation in sample classification across different values of k. **(F)** PCA plot illustrating the distribution of AAA samples. **(G)** Expression of senescence-related AAA biomarkers in groups A and B. CDF, cumulative distribution function.
